# Tuning of Magnetoimpedance Effect and Magnetic Properties of Fe-Rich Glass-Coated Microwires by Joule Heating

**DOI:** 10.3390/s22031053

**Published:** 2022-01-29

**Authors:** Alvaro Gonzalez, Valentina Zhukova, Paula Corte-Leon, Alexandr Chizhik, Mihail Ipatov, Juan Maria Blanco, Arcady Zhukov

**Affiliations:** 1 Department of Polymers and Advanced Materials: Physics, Chemistry and Technology, Faculty of Chemistry, University of Basque Country (UPV/EHU), 20018 San Sebastian, Spain; alvaro.gonzalezv@ehu.eus (A.G.); valentina.zhukova@ehu.es (V.Z.); paula.corte@ehu.eus (P.C.-L.); oleksandr.chyzhyk@ehu.es (A.C.); mihail.ipatov@ehu.es (M.I.); 2 Departamento de Física Aplicada, Escuela de Ingeniería de Gipuzkoa, University of Basque Country (UPV/EHU), 20018 San Sebastian, Spain; juanmaria.blanco@ehu.es; 3 IKERBASQUE, Basque Foundation for Science, 48011 Bilbao, Spain

**Keywords:** magnetic microwires, magnetic anisotropy, joule heating, giant magnetoimpedance, domain wall propagation, hysteresis loop

## Abstract

The influence of Joule heating on magnetic properties, giant magnetoimpedance (GMI) effect and domain wall (DW) dynamics of Fe_75_B_9_Si_12_C_4_ glass-coated microwires was studied. A remarkable (up to an order of magnitude) increase in GMI ratio is observed in Joule heated samples in the frequency range from 10 MHz to 1 GHz. In particular, an increase in GMI ratio, from 10% up to 140% at 200 MHz is observed in Joule heated samples. Hysteresis loops of annealed samples maintain a rectangular shape, while a slight decrease in coercivity from 93 A/m to 77 A/m, after treatment, is observed. On the other hand, a modification of MOKE hysteresis loops is observed upon Joule heating. Additionally, an improvement in DW dynamics after Joule heating is documented, achieving DW propagation velocities of up to 700 m/s. GMI ratio improvement along with the change in MOKE loops and DW dynamics improvement have been discussed considering magnetic anisotropy induced by Oersted magnetic fields in the surface layer during Joule heating and internal stress relaxation. A substantial GMI ratio improvement observed in Fe-rich Joule-heated microwires with a rectangular hysteresis loop and fast DW propagation, together with the fact that Fe is a more common and less expensive metal than Co, make them suitable for use in magnetic sensors.

## 1. Introduction

Soft magnetic materials play an increasing role in various technological applications [1,2,3,4,5]. One of the most relevant applications of soft magnetic materials involves the development of high performance magnetic sensors, magnetometers and devices [2,3,4,5,6,7,8]. A combination of magnetic softness with good physical properties (mechanical properties, corrosion resistance, biocompatibility) and low cost are other relevant features for industrial applications [9,10,11,12].

Amorphous materials prepared by rapid melt quenching can present one of the most favorable combinations of excellent magnetic softness together with good mechanical, corrosion properties and variable geometry [3,4,5,6,7,8,9,10,11,12,13,14]. These properties of amorphous materials are intrinsically related to their glassy-like structure.

One of the parameters affecting the magnetic softness of amorphous materials is the magnetostriction coefficient, *λ_s_*: vanishing *λ_s_* is one of the conditions for the magnetic softness optimization [15]. The main route for *λ_s_* tuning is determined by the appropriate selection of the chemical composition [15]: vanishing *λ_s_*-values can be obtained either in Co-rich amorphous materials in Co_1−*x*_Fe*_x_* or Co*_x_*Mn_1−*x*_ systems for 0.03 ≤ *x* ≤ 0.08 or in Ni- rich Fe*_x_*Ni_1−*x*_ amorphous materials for *x* ≈ 0.1 [15,16,17,18]. Most attention is paid to Co-rich amorphous materials, since Ni-rich amorphous alloys show low saturation magnetization [15].

Magnetic softness is linked to the giant magnetoimpedance (GMI) effect, consisting of an impedance dependence on the applied magnetic field [19,20,21,22]. The main features of the GMI effect are satisfactorily explained in terms of electrodynamics considering the magnetic field dependence of skin depth, *δ*, in a soft magnetic conductor [19,20,21,22]. High magnetic permeability, typically observed in amorphous materials with vanishing *λ_s_*, is one of the pre-requisites for bigger GMI effect [19,20,21,22].

One of the most prospective materials is soft magnetic wire: GMI effect has been first reported in crystalline permalloy wire with high permeability [23] and rediscovered in 90-s in amorphous Co-rich wires with nearly-zero *λ_s_* [19,20].

In magnetic wires with high circumferential magnetic permeability, *μ_ϕ_*, the dependence of the skin depth, *δ*, on applied magnetic field, *H*, is given as [19,20,21,22]:

(1) $$\delta =\;\frac{1}{\sqrt{{\pi \sigma \;\mu }_{\varphi }\;f}\;}$$

where *σ* is the electrical conductivity and *f*—the AC current frequency.

Usually the GMI effect is expressed in terms of the GMI ratio, Δ*Z*/*Z,* defined as [19,20,21,22]:

Δ*Z*/*Z* = [*Z* (*H*) − *Z* (*H_max_*)]/*Z* (*H_max_*)] × 100 (2)

where *H_max_* is the maximum applied *DC* magnetic field (as a rule below a few kA/m).

The highest GMI ratio (up to 650%) with magnetic field sensitivities up to 10%/A/m is reported in properly processed amorphous wires [21,24,25,26].

Such excellent features of GMI effect realized in amorphous wires have been employed for development of a number of high-performance magnetic sensors suitable for various technological applications [8,27,28,29,30].

However, Co belongs to the list of critical elements [31]. Therefore, search for routes allowing improvement of magnetic softness of less-expensive Fe-rich amorphous materials attract a certain attention [32,33,34].

It is worth mentioning that magnetic wires can present other unusual magnetic properties, such as spontaneous magnetic bistability associated with magnetization reversal through a large and single Barkhausen jump [35,36,37,38]. A demagnetized state in such magnetically bistable amorphous wires cannot be achieved and fast magnetization switching between two states with remanent magnetization runs through a single domain wall (DW) propagation [36,37,38,39,40]. The DW velocity, *v*, in such magnetically bistable amorphous wires can reach values above 1 km/s, making them attractive for spintronic applications [41,42].

There are several preparation methods involving rapid melt quenching suitable for the fabrication of magnetic wires with amorphous structure, such as in-rotating water, melt-extraction and Taylor–Ulitovsky methods [37,43,44,45,46]. The latter allows preparation of long (up to 10 km) amorphous microwires with the most extended diameters range (from 0.2 to 100 μm continuous microwires), covered with an insulating, flexible and biocompatible glass coating [46,47,48,49]. Excellent magnetic softness and high GMI effect have been reported in Co-rich microwires [25,26]. Consequently, several high-performance devices, techniques and sensors have been developed utilizing glass-coated magnetic microwires [50,51,52,53,54,55,56].

Outstanding magnetic properties of as-prepared amorphous microwires are further improved by appropriate postprocessing including different types of thermal treatments [25,26,57,58,59,60]. Thus, magnetic softness, GMI ratio and even DW dynamics of Fe and Co-rich microwires can be substantially improved by stress-annealing [57,58,59,61,62,63,64,65,66]. However, conventional furnace annealing is not efficient enough even for Co-rich microwires: the hysteresis loops of annealed Co-rich microwires turn into rectangular ones [45,67], while the rectangular loops character of Fe-rich microwires remains after conventional annealing [68,69,70]. On the other hand, there is a substantial improvement in DW dynamics (increase in DW velocity and DW mobility) in Fe-rich microwires after conventional annealing, achieving DW velocities of up to ~2.4 km/s [39,71]. Additionally, even higher DW dynamics improvement in stress-annealed Fe-rich microwires, up to ~3 km/s, has been attributed to the transverse character of the stress-annealing induced magnetic anisotropy [39,71,72].

On the other hand, Joule heating is an alternative technique that allowed considerable GMI ratio improvement of Co-rich microwires [25,26,66,67]. Joule heating is a fairly simple and convenient method for annealing amorphous materials. The beneficial influence of Joule heating is explained by sample heating itself as well as by circumferential Oersted magnetic field [25,67]. Although the beneficial effect of Joule heating on GMI effect of Co-rich microwires is demonstrated in several publications, the applicability of Joule heating for GMI effect and DW dynamics optimization in Fe-rich microwires has not been reported.

Consequently, in this paper we report the effect of Joule heating on magnetic properties and GMI effect of Fe_75_B_9_Si_12_C_4_ microwires.

## 2. Materials and Methods

The studied Fe_75_B_9_Si_12_C_4_ (inner metallic nucleus diameter *d* = 15.2 µm, total microwire diameter *D* = 17.2 µm) glass-coated microwires have been prepared using the Taylor–Ulitovsky technique, involving rapid melt quenching of metallic alloys covered by an insulating glass shell. As-prepared, annealed and stress-annealed microwires with said chemical composition and dimensions have already been studied in some of our previous works [34,35,57,58,59]. Therefore, we have continued their study with the aim to evaluate the influence of Joule heating on magnetic properties, GMI effect and domain wall dynamics and compare with the same properties of as-prepared, annealed and stress-annealed Fe_75_B_9_Si_12_C_4_ microwires. Details of the preparation technique are described elsewhere [46].

Hysteresis loops of as-prepared and annealed microwires were measured by the fluxmetric method previously described elsewhere [63]. An axial magnetic field, *H*, is generated by 120 mm long and thin (8 mm in diameter) solenoid. Such long, thin solenoid creates a rather homogeneous magnetic field along the axis in a long enough area inside it [38]. The magnetic field homogeneity was checked using the GM05 Gaussmeter and probe (Hirst Magnetic Instruments Ltd., Falmouth, UK). Hysteresis loops are represented as the normalized magnetization, *M*/*M_o_* (where *M_o_* is the magnetic moment of the sample at the maximum magnetic field amplitude, *H_o_*), versus the magnetic field, *H*.

The DW dynamics has been studied with a modified Sixtus Tonks set-up described elsewhere [39]. Studied microwire samples (10 cm long, the same used for measurement of hysteresis loops) were placed coaxially inside of three pick-up coils. Magnetic field was generated by a 140 mm long solenoid (10 mm in diameter) producing rather homogeneous axial magnetic field in a long enough area inside it [38]. As in the case of the fluxmetric method, the magnetic field homogeneity was checked using the GM05 Gaussmeter and probe. The DW travelling along the sample induces an electromotive force (*EMF*) in the pick-up coils recorded by an oscilloscope. The DW velocity, *v*, was estimated as:

*υ* = *l*/Δ*t* (3)

where *l* is the distance between pick-up coils and Δ*t* is the time difference between the maximum in the induced EMF.

The impedance, *Z*, of the sample and *Z*(*H*) dependencies were measured in a wide frequency range (10–100 MHz) using a vector network analyzer N5230A (VNA). As-prepared and annealed 1 cm long samples were placed in a micro-strip sample holder and impedance was obtained from the reflection coefficient *S*_11_ as described elsewhere [73,74]. A homogenous magnetic field, *H*, parallel to the microwire axis, up to 20 kA/m is created by a long solenoid. The Δ*Z*/*Z*(*H*) dependencies were evaluated from *Z*-values obtained for different magnetic fields, *H*, using Equation (2). The frequency, *f*, dependence of the GMI effect was evaluated from the dependence of a maximum GMI ratio, Δ*Z*/*Z_max_*, defined as a maximum Δ*Z*/*Z*, versus *f*.

The Joule heating was carried out by flowing a DC electrical current, *I*, through 7 cm long microwires. Joule heating conditions were selected considering that the crystallization of glass-coated microwire was previously observed at current densities, *j*, above 200 A/mm^2^ [66,75]. Accordingly, we used *I*-values from 3 up to 20 mA (16.5 ≤ *j* ≤ 110 A/mm^2^), and annealing times, *t*, up to 20 min. To exclude the effect of possible sample inhomogeneities, the effect of Joule heating was measured for five separate samples (10 cm long), each one treated with their corresponding *I*-value for equal set times. The measurement process was as follows: after annealing a sample with its designated *I*-value for a time *t*_1_ (3 min for example as the first step), we measured both the hysteresis loops and the DW dynamics, after which the same sample was annealed again, with the same *I*-value and a new time *t*_2_ to achieve the next annealing set time (for example, after 3 min at the first set, 2 min to achieve a total of 5 min of annealing). Joule heating was perfomed in air, as the insulating and continuous glass coating protects the metallic nucleus from oxidation.

On the other hand, while it would have been preferable to carry out the GMI measurements using these same samples at each annealing step, it proved impossible, as it required 1 cm long samples to be soldered to a sample holder, which would have required to cut out 1 cm pieces from our 10 cm samples after each step, eventually making them too short for DW analysis. As such, for GMI analysis, we first completed the hysteresis and DW studies, then cut 1 cm pieces from each sample to measure their GMI ratios. To complement these results, which were all for *t* = 20 min with different *I*-values, a new set of samples, each annealed with *I* = 20 mA for different times, was prepared and had their GMI measured.

The surface magnetization process has been studied by longitudinal magneto-optical Kerr effect (MOKE) described in detail elsewhere [76]. In the MOKE experiment, a polarised light from a He–Ne laser (beam diameter about 0.8 mm) was reflected on the sample surface and captured by the detector. The rotation of the light polarization angle reflected from the sample surface is proportional to the magnetization parallel to the plane of the light and the axis of the microwire. A pair of Helmholtz coils (diameter of the coils 300 mm, distance between coils of 150 mm) provided a uniform external magnetic field, *H*, with an axial orientation at the microwire. The coils were powered using a bipolar power supply that generates a magnetic field. The magnetic field was calibrated using the GM05 Gaussmeter and probe. The maximum *H*-value was 2400 A/m.

## 3. Results and Discussion

A substantial GMI ratio improvement by Joule heating.

Evidence of transverse induced magnetic anisotropy in the surface layer from GMI response and MOKE loops.

**Figure 1:**
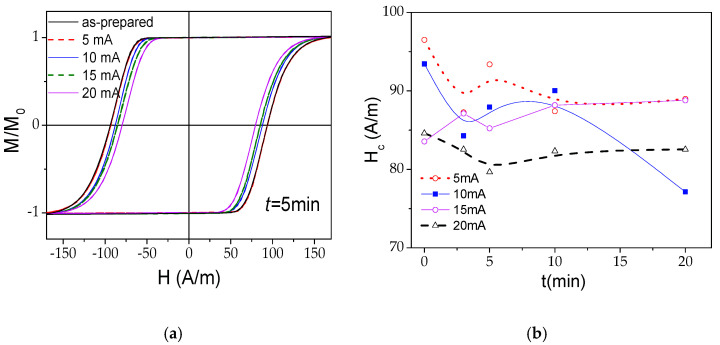
Hysteresis loops of as-prepared and annealed for *t* = 5 min for different *I* samples (**a**) and *H_c_*(*t*) dependences for the Joule heated samples with different *I*-values (**b**).

Perfectly rectangular hysteresis loops are observed in as-prepared and even Joule heated microwires (see Figure 1a), while a tendency towards a slight decrease in the coercivity, *H_c_*, can be seen, the maximum decrease being from 93 to 77 A/m for the sample annealed with *I* = 10 mA (see Figure 1b). Such hysteresis loops are typical for Fe-rich microwires with high and positive *λ_s_* (*λ_s_* ≈ 40 × 10^−6^).

The rectangular hysteresis loop observed in as-prepared and annealed microwires (see Figure 1a) is commonly explained by the particular domain structure of the Fe-rich microwires consisting of an axially magnetized single-domain inner core and an external domain shell with transverse (either radial or circular) magnetization orientation [35,36,37]. Slight decrease in *H_c_* can be attributed to internal stresses, *σ_i_*, relaxation upon annealing. Indeed, stress dependence of *H_c_* was interpreted considering that the *H_c_* is proportional to the energy required to form the DW, *γ*, involved in the bistable magnetization process [56], given as:

(4) $$H_{S}\propto \gamma \propto \frac{{\left[A\left(3/2\right)\;\lambda_{S\;}\left(\sigma +\sigma_{i}\right)\right]}^{1/2}}{cos\alpha }$$

*α* being the angle between magnetization and axial direction, *A* the exchange energy constant, *σ* the applied stresses [50]. Consequently, if *σ* = 0, *H_c_* must be affected by *σ_i_* relaxation. In amorphous materials, the magnetoelastic anisotropy, *K_me_*, and the shape anisotropy, are the main factors that determine the hysteresis loops character. *K_me_* is given by [35,36,37,56]:

*K_me_* ≈ 3/2*λ_s_*(*σ* + ≈_*i*_) (5)

The decrease in *K_me_* upon annealing must be associated with a decrease in the magnetization work and magnetic anisotropy energy, similarly to what was observed earlier in Co-rich microwires [25].

**Figure 2:**
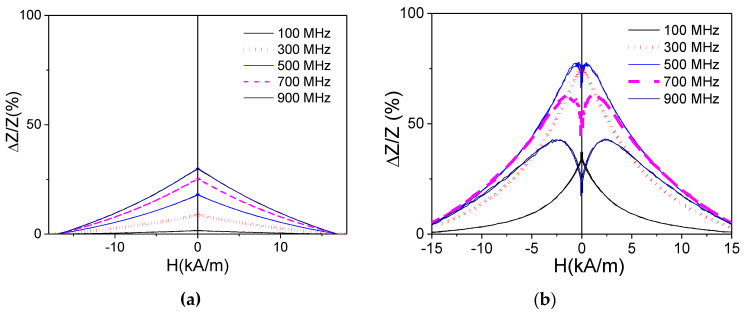
Δ*Z*/*Z*(*H*) dependencies of as-prepared (**a**) and Joule heated at *I* = 20 mA for *t* = 5 min (**b**) samples.

As expected, a rather poor GMI effect is observed in as-prepared samples, their maximum GMI ratio Δ*Z*/*Z_max_*, defined as their highest GMI max value for a set of measured frequencies, being below 30% (Figure 2a). In spite of the rectangular character of their hysteresis loops, a substantial increase in Δ*Z*/*Z* ratio is documented in a whole *f*-range in Joule heated samples (see Figure 2a,b).

**Figure 3:**
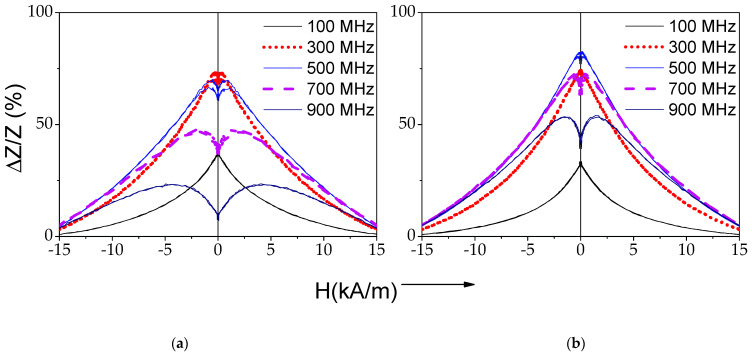
Δ*Z*/*Z*(*H*) dependencies of Joule heated at *I* = 20 mA for *t* = 10 min (**a**) and 20 min (**b**) samples.

**Figure 4:**
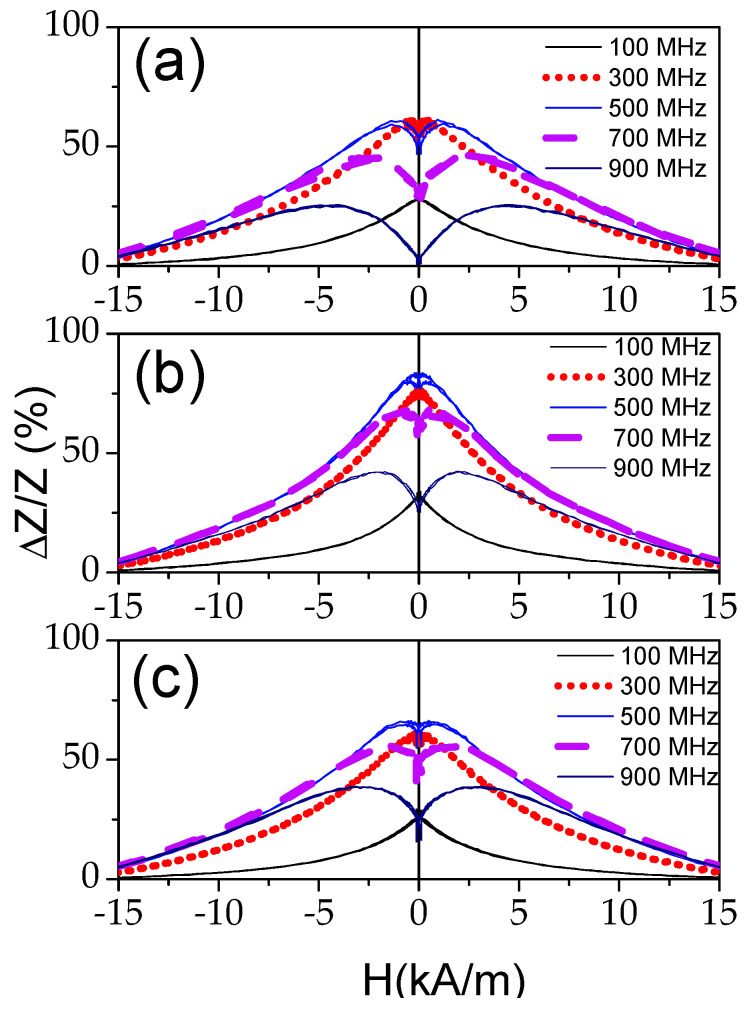
Δ*Z*/*Z*(*H*) dependencies of Joule heated for *t* = 20 min at *I =* 10 mA (**a**), *I =* 15 mA (**b**) and *I =* 20 mA (**c**) microwires.

All processed samples share two common features. The first, in all processed samples, Δ*Z*/*Z_max_* is always at least 70%. The second one, the appearance of double peak Δ*Z*/*Z*(*H*) dependencies at *f* ≥ 500 MHz (see Figure 3 and Figure 4).

Indeed, the shape of Δ*Z*/*Z*(*H*) dependencies after Joule heating becomes rather different from that of as-prepared samples. As shown in Figure 2a, a single peak Δ*Z*/*Z*(*H*) dependence is observed in as-prepared sample in a whole *f*–range; while double-peak Δ*Z*/*Z*(*H*) dependences are recorded for *f* ≥ 300 MHz in the samples Joule heated at *I =* 20 mA (5 ≤ *t ≤* 20 min) (Figure 2b and Figure 3). Similar Δ*Z*/*Z*(*H*) dependences are observed for the samples Joule heated at different *I*-values (*t* = 20 min) (see Figure 4).

**Figure 5:**
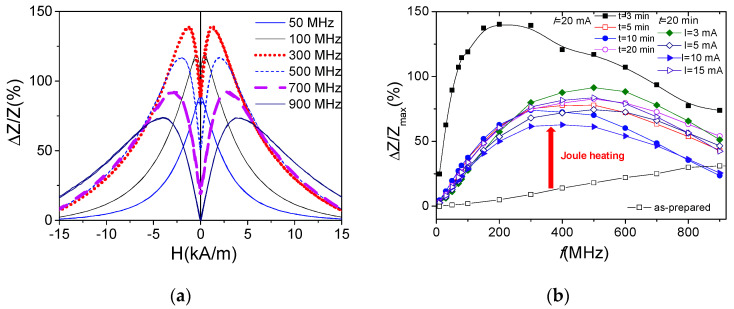
Δ*Z*/*Z*(*H*) dependencies of Joule heated for *t* = 3min at *I =* 20 mA sample (**a**) and *Z*/*Z_max_*(*f*) dependencies of as-prepared and Joule heated samples (**b**).

The most singular of Δ*Z*/*Z*(*H*) dependencies is observed in the sample processed by Joule heating at *I =* 20 mA (*t* = 3 min), as a value of Δ*Z*/*Z_max_* ≈ 140% is achieved (see Figure 5a). Additionally, double-peak Δ*Z*/*Z*(*H*) dependencies are observed starting from lower frequencies (*f* ≥100 MHz).

Previously, the influence of Joule heating on GMI effect has been studied only for Co-rich microwires with vanishing *λ_s_* [25,26]. It is worth mentioning that good magnetic softness and high GMI ratio is usually observed even in as-prepared Co-rich microwires with vanishing *λ_s_*, while Joule heating allowed further GMI ratio improvement (up to 650%) [25,26]. Additionally, a double-peak Δ*Z*/*Z*(*H*) dependence was observed in both as-prepared and Joule heated Co-rich microwires [25,26].

In contrast, rather poor GMI ratio and a single-peak Δ*Z*/*Z*(*H*) dependence are observed in as-prepared Fe-rich microwires (Figure 2a). Such difference is commonly attributed to rather low circumferential magnetic permeability of Fe-rich microwires with rectangular hysteresis loops and axial character of magnetic anisotropy. After Joule heating, a substantial GMI ratio improvement (almost an order of magnitude) and change of the Δ*Z*/*Z*(*H*) dependence character from a single-peak to a double-peak Δ*Z*/*Z*(*H*) dependence are recorded (see Figure 2b, Figure 3, Figure 4 and Figure 5).

**Figure 6:**
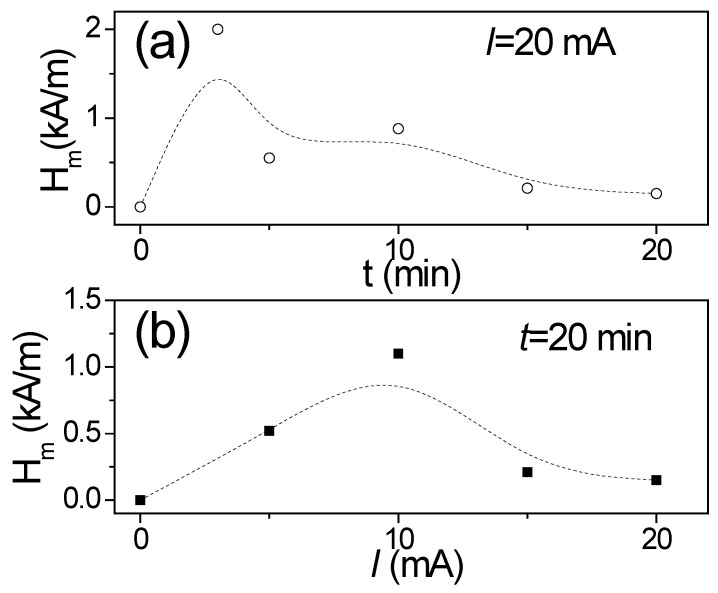
*H_m_* dependencies on *t* (**a**) and on *I* (**b**) evaluated from Δ*Z*/*Z*(*H*) dependencies at *f* = 500 MHz. The lines are just guides for the eyes.

Generally, a double-peak Δ*Z*/*Z*(*H*) dependence in magnetic wires is attributed to the circumferential character of magnetic anisotropy, in contrast to single-peak Δ*Z*/*Z*(*H*) dependence predicted for magnetic wires with axial magnetic anisotropy [21,77]. Additionally, the magnetic field of the maximum in Δ*Z*/*Z*(*H*) dependencies is associated to the magnetic anisotropy field, *H_m_*, [21,22,77]. Therefore, the observed transformation of Δ*Z*/*Z*(*H*) dependence upon Joule heating must be attributed to a change of the character of magnetic anisotropy from axial to transverse. However, only a slight modification of hysteresis loop consisting in a slight decrease in *H_c_* upon Joule heating is observed (see Figure 1). The shape of the hysteresis loops of as-prepared and Joule heated samples is quite similar. The origin of such a discrepancy can be understood considering that the GMI effect involves only the sample portion within the skin depth. From measured Δ*Z*/*Z*(*H*) dependences, we tried to evaluate the modification of *H_m_* versus *I* or *t*. For this evaluation, we selected *f* = 500 MHz, considering that at 500 MHz, all annealed samples present double-peak Δ*Z*/*Z*(*H*) dependence. As can be seen from Figure 6a,b, there is an increase in *H_m_*, followed by a decrease, with an increase in either *t* or *I.*

The magnetization distribution of magnetic wires is commonly discussed considering the contribution of the exchange energy tending the axial magnetization alignment, magnetoelastic and induced magnetic anisotropies [67,68,70,78]. The peculiarity of Joule heating is that it involves sample heating in the presence of a circumferential magnetic field, *H_circ_*, created by a current flowing through the sample [26,67], given as:

*H_circ_* = *Ir*/2*πR*^2^ (6)

where *r* is radial distance, *R*- microwire metallic nucleus radius.

Accordingly, *H_circ_* varies from zero on the microwire axis to the maximum value (*H_circ_* ≈ 0.43 kA/m for *I* = 20 mA) on the metallic nucleus surface. This *H_circ_*-value at the surface is superior to the *H_c_*-values obtained from the hysteresis loops (see Figure 1a). Taking into account that the surface layer of the magnetic microwire is magnetized by *H_circ_*, during Joule heating, one can expect the appearance of induced magnetic anisotropy on the surface. Consequently, the evolution of magnetic anisotropy, as evidenced by *H_m_*(*t*) (Figure 6a) and *H_m_*(*I*) (Figure 6b) dependencies, can be associated to the counterbalance between the shape, magnetoelastic and induced anisotropies.

Domain structures of magnetic wires are commonly described in terms of a core-shell model [36,37,78,79]. In terms of such a model, the domain structure of magnetically bistable microwires is described as consisting of a single axially magnetized domain surrounded by an outer domain shell with transverse (either radial or circumferential) magnetization. The inner, axially magnetized core radius, *R_ic_*, can be estimated from the remanent magnetization, *M_r_*/*M_o_* as [36,37,78,79]:

*R_ic_* = *R*(*M_r_*/*M_o_*)^l/2^ (7)

In the present case, *M_r_*/*M_o_*, evaluated from the hysteresis loops provided in Figure 1a, gives the values *M_r_*/*M_o_ ≈* 0.95. Therefore, the thickness of the outer domain shell, *t_OD_*, estimated as the difference between *R* and *R_ic_*, gives values of about 0.2 μm. Such values are comparable with the skin depth, *δ*, values estimated for *f* = 500 MHz [80].

**Figure 7:**
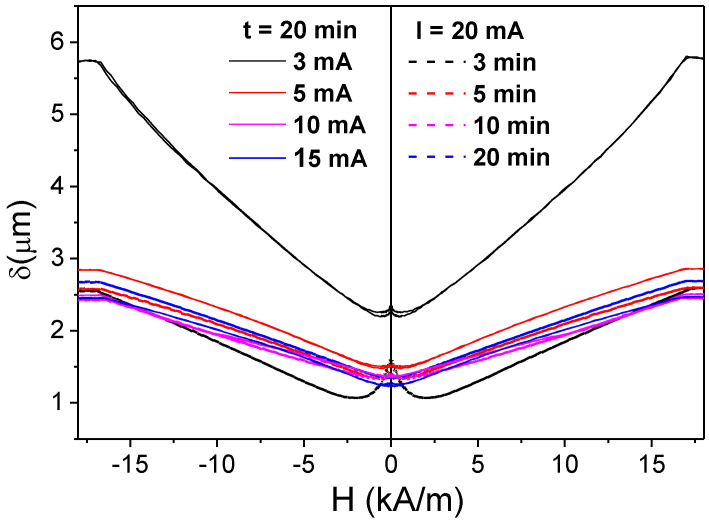
Skin depth, *δ*(*H*), evaluated with Equation (6) for all samples at *f* = 500 MHz.

The penetration depth, *δ*, can be evaluated from the Δ*Z*/*Z*(*H*) dependences assuming that that the *Z*(*H*) dependence is attributed to changes in *δ* [81]. In this assumption, *δ* can be calculated as:

*δ = R* [1 − (1 − *R_DC_*/*R_AC_*)^1/2^] (8)

where *R_DC_* is the sample DC resistance, and *R_AC_*, the real part of its impedance, *Z*(*H*). As such, we obtained minimum skin depth values, *δ_min_*, at *f* = 500 MHz of about 1 µm (see Figure 7) for most samples, the only exception being the *I =* 3 mA*, t =* 20 min sample which shows *δ_min_* values up to nearly 2.5 µm.

Obtained *δ_min_* values are comparable with the *t_OD_* evaluated from Equation (5). Consequently, the outer domain shell with transverse magnetic anisotropy can substantially affect the GMI response at a sufficiently high frequency (i.e., at *f* = 500 MHz). Indeed, the GMI effect features are linked to magnetic properties of the surface layer. Given the provided above results, that bulk hysteresis loops character of Joule heated samples remain almost the same (see Figure 1), and we can assume that such modification of magnetic anisotropy occurs only in the surface layer of metallic nucleus.

**Figure 8:**
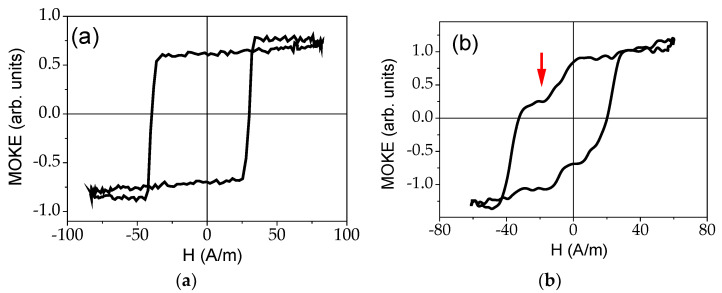
MOKE hysteresis of as-prepared (**a**) and Joule heated (**b**) microwire.

The MOKE technique can provide additional information of surface magnetic properties. Similarly to bulk hysteresis loops, an almost perfectly rectangular MOKE hysteresis loop is observed in the as-prepared sample (see Figure 8a). Such character of hysteresis loops must be related to the axial magnetic bistability.

The MOKE hysteresis loop of Joule heated samples is rather different (see Figure 8b). Magnetic bi-stability is no longer observed. The hysteresis shape can be associated with the formation of a metastable magnetic state close to the circular direction of the magnetization (marked by the red arrow).

**Figure 9:**
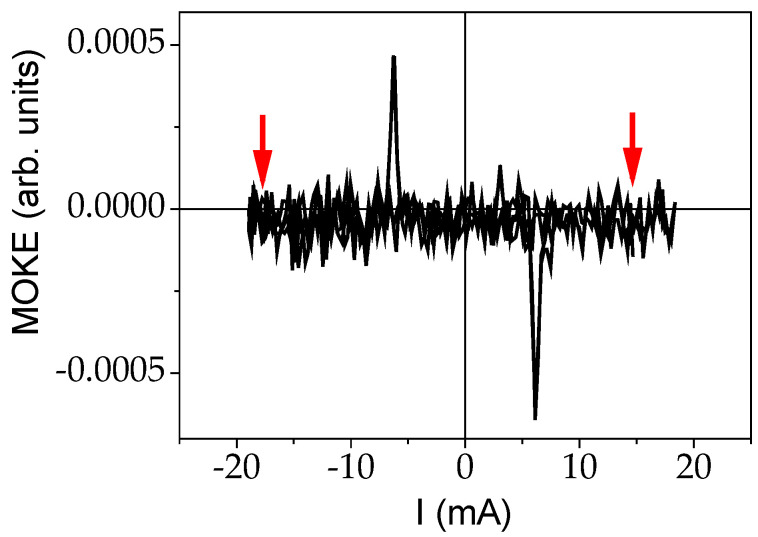
MOKE hysteresis of Joule heated microwire obtained in a circular magnetic field.

The MOKE signal measured versus electric current flowing along the microwire (proportional to the circular magnetic field) is provided in Figure 9. Two pronounced peaks are observed. In the presence of a circular magnetic field, the magnetization reversal occurs between two circular states. Given that we are using longitudinal MOKE, the two circular states give “0” MOKE signal (marked by red arrows). The two observed peaks must be associated with sharp jumps between two circular (or inclined) states, in other words, the effect of circular magnetic bistability, as previously described [82].

Accordingly, from comparative analysis of GMI response and MOKE loops we can deduce the induced magnetic anisotropy of transverse character in the surface area of the samples processed by Joule heating.

**Figure 10:**
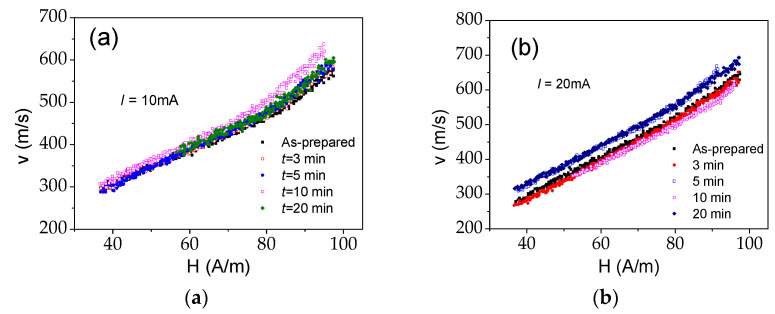
*v*(*H*) dependencies measured in as-prepared and Joule heated samples at *I* = 10 mA (**a**) and 20 mA (**b**).

The perfectly rectangular character of hysteresis loops of as-prepared and all Joule heated samples (see Figure 1a) suggests that the remagnetization process of all the samples runs through a single DW propagation. Accordingly, we measured *v*(*H*) dependencies of as-prepared and Joule heated samples. As can be observed from Figure 10, generally, Joule heating allows DW velocity improvement.

It is worth noting that Fe-rich microwires, Joule heated at appropriate conditions, have a unique combination of magnetic properties: they exhibit a fast single DW propagation, together with high GMI effect in the same microwire. Recently, such a unique combination of high GMI effect and fast DW propagation was observed in stress-annealed Fe-rich microwires for certain stress-annealing conditions [58,65,83,84].

Nearly linear *v*(*H*) dependencies are observed in as-prepared and Joule heated samples (see Figure 10). As explained [36,40,43], linear *v*(*H*) dependencies are attributed to a viscous regime, described as:

*ν* = *S* (*H* − *H*_0_) (9)

where *S* is the DW mobility and *H*_0_ is the critical propagation field, below which the DW propagation is not possible.

Previously, an increase in DW velocity and DW mobility observed in Fe-rich microwires after annealing is discussed in terms of the relationship between the DW mobility, *S*, and *K_me_* and the internal stresses relaxation [39,71].

On the other hand, DW dynamics improvement in various magnetic wires has been achieved under application of transversal bias magnetic field [85,86,87,88].

The transverse induced magnetic anisotropy in the surface layer of the samples processed by Joule heating is evidenced by MOKE experiments. It was recently found that the transverse magnetic anisotropy affects a travelling DW in the same way as the application of a transversal bias magnetic field [72]. Accordingly, observed DW dynamics improvement can be attributed to the transverse magnetic anisotropy in the outer domain shell associated to Joule heating as well as to internal stresses relaxation during Joule heating. To understand the origin for the DW dynamics improvement, we must consider that annealing as well as induced magnetic anisotropy affect the DW dynamics of glass-coated microwires [71,72].

However, the observed DW dynamics improvement is less pronounced than for Fe-rich microwires subjected to conventional furnace annealing [39].

The origin of the magnetic field annealing induced anisotropy is commonly attributed to the either directional atomic pair ordering or a single–atom mechanism considering a dense-random-packing-of-hard-spheres-like structure of amorphous materials with B occupying interstitial holes [89,90,91].

## 4. Conclusions

Joule heating allows a substantial GMI ratio improvement, from 30% to 140% Δ*Z*/*Z_max_*, of Fe_75_B_9_Si_12_C_4_ glass-coated microwires. On the other hand, only a slight decrease in coercivity of up to 16 A/m is observed, while hysteresis loops of Joule heated samples remain rectangular. Single domain wall propagation is observed in as-prepared and Joule heated microwires. After Joule heating, an improvement of domain wall dynamics is achieved, with do71main wall velocities of up to 700 m/s. The observed GMI ratio improvement, modification of MOKE hysteresis loops and domain wall dynamics have been discussed considering magnetic anisotropy induced by Oersted magnetic field in the surface layer of microwire during Joule heating and internal stress relaxation. Substantial GMI ratio improvement observed in Joule-heated Fe-rich microwires presenting bulk rectangular hysteresis loop and fast DW propagation, along with the fact that Fe is a more abundant and less expensive metal than Co, makes them suitable for various technological applications including magnetic sensors, smart composite materials, non-destructive and non-contact monitoring of the composites. Fe-rich microwires, current annealed under appropriate conditions, exhibit a unique combination of magnetic properties such as a fast single DW propagation, together with a high GMI effect in the same microwire.

## Data Availability

Data available on request due to restrictions related to the developing projects.
